# A Flexible and Attachable Colorimetric Film Sensor for the Detection of Gaseous Ammonia

**DOI:** 10.3390/bios12080664

**Published:** 2022-08-21

**Authors:** Sangwon Lee, Eun-Hee Lee, Seung-Woo Lee

**Affiliations:** 1 Department of Fine Chemistry, Seoul National University of Science and Technology, 232 Gongneung-ro, Nowon-gu, Seoul 01811, Korea; 2 Department of Microbiology, Pusan National University, 2 Busandaehak-ro 63 Beon-gil, Geumjeong-gu, Busan 46241, Korea; 3 Department of Nano Bio Engineering, Seoul National University of Science and Technology, 232 Gongneung-ro, Nowon-gu, Seoul 01811, Korea; 4 Center for Functional Biomaterials, Seoul National University of Science and Technology, 232 Gongneung-ro, Nowon-gu, Seoul 01811, Korea

**Keywords:** toxic gas, NH_3_, optical detection, low cost, occupational safety

## Abstract

A cost-effective, simple, flexible, and disposable colorimetric film sensor was constructed for the rapid detection of gaseous ammonia. The sensor was designed to consist of three layers, namely top, middle, and bottom layers of a polymeric elastomer. The bromocresol (BCG) indicator embedded in the middle layer of the film facilitated a change in color of the sensor from yellow-orange to blue upon exposure to gaseous ammonia. The color change was visually observed by the naked eye. The sensitivity of the sensor was verified by a successful detection of gaseous ammonia at concentrations from 4 to 235 ppm within 3 min, and the corresponding visual detection of ammonia gas was at a concentration as low as 11 ppm. The sensor also achieved a selective detection of gaseous ammonia over a variety of alkaline chemicals. The color of the sensor exposed to ammonia reverted from blue to the original yellow-orange upon subsequent exposure to the fume of acetic acid or aeration for 48 h, and it showed reliable performance for the detection of gaseous ammonia even after five repeated uses. The applicability of the sensor was validated by attaching it onto a safety helmet for a simulation of an industrial ammonia gas leak. The results indicated that our colorimetric film sensor is affordable, disposable, and reproducible, and can serve as an effective alternative for simple and rapid recognition of gaseous ammonia in environmental and air quality monitoring as well as in industrial applications.

## 1. Introduction

Ammonia (NH_3_) serves as a precursor for amino acid and nucleotide syntheses and is an important source of nitrogen for growing plants. In nature, ammonia is produced via bacterial processes associated with the biogeochemical nitrogen (N) cycle. Ruminant digestion is one of the largest sources of ammonia production in natural environments [1]. In industry, the global production of ammonia was estimated to be 235 million metric tons in 2019 and is expected to expand to nearly 290 million metric tons by 2030 [2]. Nearly 80% of produced ammonia is used for the synthesis of fertilizer in agriculture, and such fertilizer is thought to be responsible for 50% of global food production. Ammonia is also found in pharmaceuticals, refrigerant gas, cleaning solutions, plastics, textiles, and explosives, and is recognized as a sustainable and renewable energy fuel [3,4,5,6,7]. Ammonia emission mostly originates from areas of intense agricultural, domestic, and industrial activities and these anthropogenic emissions dominate natural sources [8,9]. The widespread use of ammonia leads to people being exposed to ammonia from inhalation of the gas or vapors and has implications for human health and life expectancy.

Accidental release of ammonia gas often occurs during manufacture, use, and transportation, and has resulted in life-threatening situations. Recently, BQ prime (powered by Bloomberg) reported that an incident of ammonia gas leakage from the Indian Farmers Fertilizer Cooperative (IFFCO) fertilizer plant in Uttar Pradesh killed two IFFCO officials and led to the hospitalization of sixteen people [10]. Therefore, it is highly important to regularly monitor ammonia gas, so that any such gas leaks can be recognized and that prompt and appropriate actions can be taken.

Ammonia is a colorless, corrosive, explosive, and toxic agent, and has an unpleasant pungent odor. It is well known for its detrimental effects on human health such as severe skin irritation and eye damage [11]. Lung disorders and permanent blindness can be caused by inhaling a high concentration of ammonia. The Occupational Safety and Health Administration (OSHA) sets the workplace exposure limit for gaseous ammonia in the United States, and has specifically set the legal airborne permissible exposure limit (PEL) to be 50 ppm averaged over an 8 h work shift. [11,12]. Persons exposed to higher levels become uncomfortable due to the odor and irritation caused by the ammonia. The pungent smell of ammonia allows its presence to be recognized at concentrations as low as 5 ppm in the air. However, sensitivity to smells differs between people and odor thresholds vary greatly [11] because prolonged exposure to gaseous ammonia even at low concentrations causes olfactory fatigue or adaptation [13]. As a result, those exposed to ammonia for a long time, such as workers in industrial settings, lose awareness of the pungent smell [13,14]. Therefore, alarm and warning systems for gaseous ammonia are needed in industrial facilities having on-site workers, and need to operate with a detection limit of 3~50 ppm and a response time of minutes [15,16].

Various sensing techniques have been developed for the detection of ammonia gas [17,18,19,20,21,22,23,24]. Gas sensors using laser-coupled spectroscopy and photoacoustic spectroscopy are applicable in diverse industries, especially in ammonia production plants where sensitive and continuous gaseous ammonia sensing performance is required. Metal-oxide-based gas sensors have received attention due to the advantages of simplicity and good process compatibility; particularly, SnO_2_, ZnO, WO_3_, TiO_2_, and MoO_3_ are widely used metal oxides in ammonia detection [17]. Chemiresistors using a conducting polymer offer the advantage of quick response time with a relatively low limit of detection (<10 ppm) [17,25]. Despite their advantages of high sensitivity and continuous monitoring functions, these techniques suffer from drawbacks such as high cost and the need to use sophisticated instruments operated by skilled personnel, and are, hence, generally unsuitable for on-site workers in small ammonia production facilities. For these and other cases, it is necessary to develop and implement a low-cost, simple, and disposable sensor.

In this study, a flexible, easily attachable, and disposable colorimetric film sensor was designed for the detection of ammonia gas. The sensor consisted of three film layers. Its physical, chemical, and manufacture properties including permeability, stretchability, reproducibility, and scalability were analyzed. For simplicity, cost-effective colorimetric detection was employed in the sensor design, allowing for visual observation of detection by the naked eye. Upon exposure to ammonia, the sensor exhibited a significant change in color from yellow-orange to blue within 3 min, and its sensitivity and selectivity specific to ammonia were validated in spectral and photographic analyses. Finally, we demonstrated the applicability of the sensor for on-site workers. As a simulation of ammonia production facilities, the sensor was attached to a safety helmet, and a change in the color of the sensor was measured within 3 min of a release of ammonia gas.

## 2. Materials and Methods

### 2.1. Reagents and Apparatus

Poly(dimethylsiloxane) (PDMS, Sylgard 184) and the curing agent were purchased from K1 solution (Gyeonggi-do, Korea). Sodium hydroxide (NaOH), potassium hydroxide (KOH), and barium hydroxide (Ba(OH)_2_) were obtained from Samchun Chemicals Co., Ltd. (Seoul, Korea). Bromocresol green sodium salt (BCG) was purchased from Sigma-Aldrich (Saint Louis, MO, USA). The concentration of gaseous ammonia was continuously measured using a portable Landfill Gas Analyzer (Geotech GA5000, QED Environmental Systems Ltd., Coventry, UK).

### 2.2. Fabrication of the PDMS-Based Colorimetric Film Sensor for Gaseous Ammonia Detection

The colorimetric film sensor we made consisted of three layers of PDMS, namely top, middle, and bottom layers. The middle layer was composed of a thin film of a mixture of PDMS and the sensing material BCG. The top and bottom layers were composed each of a thin film of PDMS to protect the sensing material.

First, the PDMS-based bottom layer was fabricated on a polystyrene Petri dish (diameter of 9 cm). Specifically, PDMS and the curing agent (10:1, *w*/*w*) were mixed using a spatula, and subjected to the action of a vacuum pump to remove bubbles. An aliquot (0.5 g) of this pre-mixed PDMS was placed at the center of the Petri dish, and spin-cast by using a spin coater (ACE-200, Dong Ah Trade Co., Seoul, Korea) at 6000 rpm for 15 s. The PDMS-coated Petri dish was transferred to a hot plate (PC-420D, Corning, New York, NY, USA) and subjected to 70 °C for 1 h for curing the film.

For the middle sensing layer, aliquots of a solution of 37.5 mg/mL of BCG in deionized (DI) water were added into respective aliquots of the above-described premixed PDMS to desired BCG concentrations of 1, 3, 5, and 10 mM. The resulting samples were each homogeneously mixed using a magnetic stirrer, and subjected to the operation of a vacuum pump to eliminate bubbles. An aliquot (0.5 g) of the BCG-PDMS mixture was placed at the center of the bottom PDMS layer on the Petri dish, and then spin-cast at 3000 rpm for 15 s. This Petri dish, i.e., that coated with both the bottom PDMS layer and middle sensing layer, was transferred to the hot plate and subjected to 70 °C for 4 h for curing the sensing layer. After this curing, the top layer was fabricated by applying the same components in the same amounts and conditions that were employed for the fabrication of the bottom PDMS layer.

### 2.3. Sensor Thickness Measurements

The thickness and uniformity levels of the sensing layer made of just the BCG-PDMS mixture (i.e., BCG-PDMS layer) and of the three layers of the colorimetric film sensor (i.e., PDMS/BCG-PDMS/PDMS) were measured using a three-dimensional (3D) surface confocal laser scanning microscope (LSM 800, ZEISS, Oberkochen, Germany). The specimens were prepared by cutting each film into 1 cm × 1 cm pieces. Each measurement was performed in triplicate.

### 2.4. Sensor Contact Angle Measurements

A colorimetric film sensor sample was cut into 1 cm × 1 cm pieces and the contact angle of these pieces with a 5 μL DI water droplet was measured at room temperature using a contact angle meter (CAM-200, KSV Instruments Ltd., Helsinki, Finland). An average of three readings on a film were taken as the water contact angle. The experiments were performed in triplicate.

### 2.5. Leaching of Bromocresol Green (BCG) Indicator from the Sensor

The leachability of BCG indicator from the PDMS-based film sensor was evaluated by measuring the levels of released BCG from two films: a sample of the BCG-PDMS film (i.e., middle sensing layer only) and a sample of the BCG-PDMS film that was sandwiched by the two PDMS protection layers (i.e., the full PDMS/BCG-PDMS/PDMS sensor). A piece (1 cm × 1 cm) of each film was separately immersed in a vial containing 2 mL of DI water and incubated at room temperature for 72 h. The resulting leachate was transferred to a quartz cuvette and its absorbance was measured at 628 nm using a flame spectrometer (Ocean Optics Inc., Orlando, FL, USA). All of the experiments were performed in triplicate.

### 2.6. Optimization of Bromocresol Green (BCG) Concentration in the Sensor

Samples of the colorimetric film sensors having different concentrations of BCG indicator, specifically 1, 3, 5, and 10 mM, were prepared. For each BCG concentration, four experiments were carried out, each with a piece of the film sensor attached to the inside of a 100 mL flask and exposed to gaseous ammonia, for ammonia concentrations of 11, 29, 100, and 239 ppm, respectively. The concentration of gaseous ammonia was measured using a gas analyzer that was connected to the flasks. The absorbances of the film sensor at 628 nm were periodically measured using a flame spectrometer. All of the experiments were performed in triplicate.

### 2.7. Sensitivity of the Sensor for the Detection of Gaseous Ammonia

To determine the sensitivity of the colorimetric film sensor for the detection of gaseous ammonia, absorbance spectra were collected. First, a piece (1 cm × 1 cm) of the sensor having a BCG indicator concentration of 5 mM was attached to the surface of a quartz cuvette, and the absorbance spectrum of this sensor (the “original” spectrum) was measured in ambient air in the wavelength range of 400–800 nm using a flame spectrometer. After acquiring this spectrum, each film patch was transferred to the inside of a respective 100 mL flask, and the flasks were filled with gaseous ammonia to concentrations of 4, 11, 21, 43, 85, 128, 170, and 235 ppm, respectively. The sensors were exposed to ammonia gas for 3 min. For a negative control, the sensor was exposed to ambient air (instead of ammonia) for 3 min. After a given incubation, with ammonia or ambient air, each sensor was transferred to a quartz cuvette and its absorbance spectrum was acquired, in the same manner as described above, to determine any spectral changes. Simultaneously, the sensors were photographed to provide a visual assessment of any changes in color. The sensitivity of the sensor was determined from the difference in the absorbance at 628 nm (Δ*A*628) between that of the original film sensor (*A*628*_O_*) and that exposed to ammonia gas (*A*628*_E_*), i.e., by using the equation

(1) $$\Delta A628=A628_{E}-A628_{O}$$

All of the experiments were performed in triplicate.

### 2.8. Selectivity of the Sensor

In each of several experiments, a piece of the film sensor (1 cm × 1 cm) containing 5 mM BCG indicator was attached to the inside of a 100 mL flask. The attached sensors were separately exposed to, respectively, ammonia (a positive control), air, argon (Ar), oxygen (O_2_), nitrogen (N_2_), and hydrogen (H_2_) gases; NaOH, KOH, and Ba(OH)_2_ for 3 min. The air, argon, oxygen, nitrogen, and hydrogen gases were introduced into the vials by purging each gas. Each alkaline chemical was introduced into the respective flask by evaporating solutions of NaOH, KOH, and Ba(OH)_2_ at a concentration of 300 ppm. Separately, the film sensor was immersed in each solution of 1 M NaOH and 250 ppm of ammonia. As a negative control, a film sample was soaked in DI water. After a given duration, the immersed film sensors were washed with DI water and dried using N_2_ gas. The spectral and colorimetric changes of the film sensor were determined as described above. All of the experiments were carried out in triplicate.

### 2.9. Application of the Colorimetric Film Sensor to the Detection of Released Ammonia Gas

For each of several experiments, a piece of the film sensor (2 cm in diameter) having a BCG indicator concentration of 5 mM was attached to the surface of an industrial safety helmet, and this helmet was then placed inside a chamber (86 × 56 × 72.5 cm, width × length × height). For the different experiments, gaseous ammonia was then introduced into the chamber to final concentrations of 0 (a negative control), 17, 47, 100, 207, and 457 ppm, and in each case, the helmet was exposed to the gaseous ammonia for 3 min. After this exposure, the helmets were photographed to observe any changes in the colors of the attached sensor. For absorbance spectral measurements, the sensors were transferred to quartz cuvettes and their absorbances were recorded at 628 nm. The performance of the sensor was determined from the difference in the absorbance at 628 nm (Δ*A*628) between that of the original film sensor and that exposed to ammonia gas as described earlier. All of the experiments were performed in triplicate.

## 3. Results and Discussion

### 3.1. Properties of the Colorimetric Film Sensor

**Figure 1:**
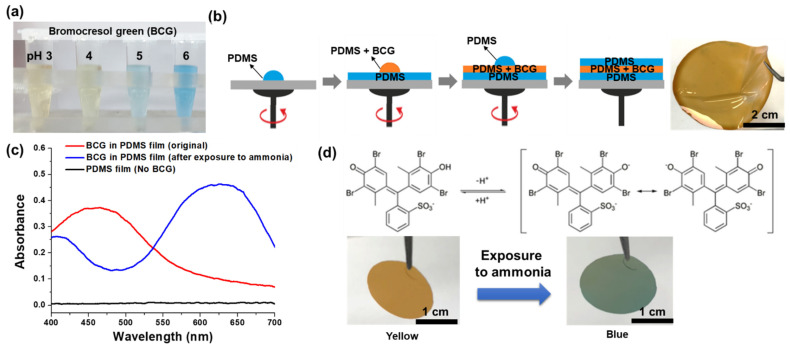
(**a**) Photographs of bromocresol green (BCG) indicator samples at different pH levels, showing different colors. (**b**) A schematic diagram of the fabrication of the colorimetric film sensor used in the current work. (**c**) Absorbance spectrum of PDMS alone, and absorbance spectra of BCG-embedded PDMS showing yellow and blue colors. (**d**) Mechanism underlying the change in color displayed by BCG molecules subjected to a changing pH environment, and optical images showing the different colors of the fabricated sensor before and after it was exposed to ammonia.

In this study, a colorimetric film sensor was designed to detect gaseous ammonia using BCG indicator incorporated into thin PDMS films. BCG is an established pH indicator that exhibits a significant change in color from yellow to blue as the pH is increased from 3 to 6 (Figure 1a) [26]. At low pH levels of 3 to 4, a strong absorption band was only observed at 400–500 nm, while an absorption band at 600–700 nm was found for pH levels higher than 5. Of the various available pH indicators, BCG was chosen due to its low pK_a_ value of 4.7. The low pK_a_ value of BCG was expected to result in a rapid response to amine-based substances such as ammonia (NH_3_), dimethyl amine (DMA), and trimethylamine (TMA), due to the pK_b_ values of these chemicals being in the range 3.2–4.8. The BCG indicator was incorporated into a silicon-based elastomer, specifically PDMS, to improve its usability in the field. As shown in Figure 1b, the colorimetric ammonia gas sensor was fabricated by spin-casting bare PDMS and a BCG-PDMS mixture. First, a bottom PDMS layer was placed on a Petri dish. Next, the BCG-incorporated PDMS layer (i.e., the sensing layer) was introduced onto the bottom PDMS layer, and then a top PDMS layer was placed onto the middle layer. This simple fabrication process was used to produce a thin and flexible colorimetric sensor on a large scale. Here, the BCG-based sensing layer was protected by the top and bottom PDMS layers. Due to the excellent optical transparency of PDMS in the visible region, no significant peaks were observed in the absorbance spectrum of the PDMS film alone (i.e., without BCG indicator, black line in Figure 1c). On the other hand, the BCG-PDMS layer, which displayed a yellow-orange color, showed a distinct absorbance spectrum, with an absorbance peak at 460 nm (red line in Figure 1c). Notably, a strong absorbance peak at 628 nm appeared when the film sensor was exposed to gaseous ammonia (blue line in Figure 1c). The significant color change in the presence of this Lewis base gaseous molecule was attributed to the occurrence of structural changes in BCG, specifically from a monoanionic form (i.e., yellow color) to dianionic form (i.e., blue color) after deprotonation by the base (Figure 1d). The dianionic form after deprotonation can be stabilized by molecular resonance: as a result, the absorption band was redshifted upon exposure to basic substances (Figure 1c) [26]. A distinct shift in color from yellow-orange (~460 nm) to blue (~628 nm) was easily recognized by the naked eye (Figure 1d).

**Figure 2:**
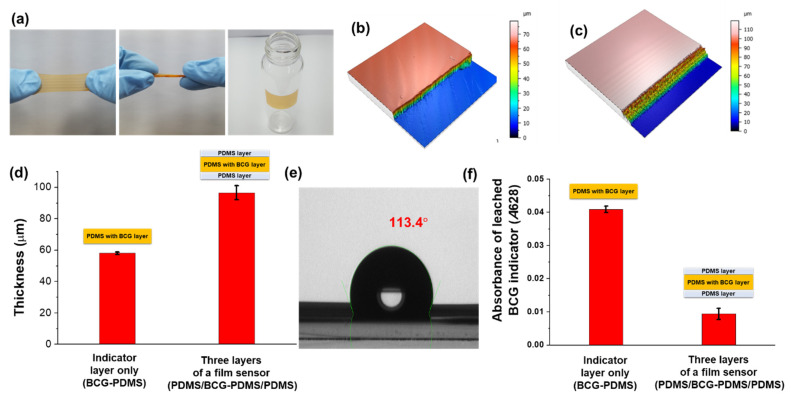
(**a**) Optical images showing the stretchability and flexibility of the designed PDMS-based colorimetric film sensor. Three-dimensional (3D) surface images of (**b**) a bromocresol green (BCG) indicator layer alone and of (**c**) the full three-layer film sensor. (**d**) Thicknesses of the BCG indicator layer alone and of the full sensor including the PDMS protection layers. (**e**) Image of DI water on the sensor, with the measured contact angle. (**f**) Levels of BCG indicator leached out from the BCG-PDMS layer with or without PDMS protection layers.

Our colorimetric film sensor was observed to be flexible, stretchable, and twistable, and could be attached to other materials (Figure 2a), attributed to PDMS having been used as the foundation material for the sensor fabrication. The properties of PDMS provided various functionalities for the sensor [27,28,29,30], with (i) the transparency of PDMS making it suitable for optical detection, (ii) its elasticity making it flexible and allowing it to be attached to other materials without becoming damaged or damaging the other materials, (iii) its permeability allowing for gas molecules to pass through it, and (iv) its inertness rendering it nonreactive toward most chemicals [30,31,32]. The spin-casting-based fabrication method resulted in a production of a uniform colorimetric sensor. The 3D images acquired of a sample sensor showed a flat surface (Figure 2b,c). The scanning electron microscope (SEM) images also showed a smooth surface of the film sensor (Figure S1 in Supplementary Information). The three layers of the film sensor together showed an overall thickness of 96.6 ± 4.5 μm, while the thickness of only the BCG indicator layer was measured to be 58.1 ± 0.8 μm (Figure 2d), indicating a thickness of 19.25 μm for each of the PDMS-based protection layers coating the BCG indicator layer. The contact angle of the sensor with DI water was measured to be about 113° (Figure 2e). The hydrophobicity of PDMS was important to make it less permeable to aqueous basic ions (e.g., hydroxide ions), which can otherwise directly react with the BCG indicator. In addition, having a hydrophobic protection layer can decrease the leachability of BCG from the sensing layer. Only a marginal amount of BCG indicator leached out from the sensor. The absorbance of BCG leachate (*A*628) from the film sensor corresponded to 0.009 ± 0.001. The absorbance of BCG leachate (*A*628) from the BCG-PDMS film (no protection layers) was estimated to be 0.041 ± 0.001, i.e., nearly four-fold higher than that from the film sensor. The insignificant leaching out of BCG indicator from the film sensor was attributable to the PDMS coatings. The impermeability of PDMS to liquid water prevented the leaching out of BCG indicator from the sensor [30].

### 3.2. Optimization of the Concentration of Bromocresol Green (BCG) Indicator in the Sensor

**Figure 3:**
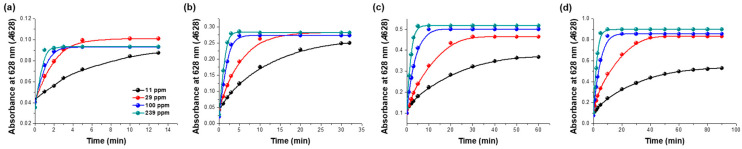
Time profiles of absorbance at 628 nm (*A*628) of colorimetric film sensors having different concentrations of the bromocresol green (BCG) indicator, specifically (**a**) 1, (**b**) 3, (**c**) 5, and (**d**) 10 mM, and exposed to the indicated concentrations of ammonia.

The colorimetric film sensors having different concentrations of BCG indicators, namely 1, 3, 5, and 10 mM, showed different absorbance changes at 628 nm upon exposure to ammonia gas (Figure 3). The film sensor with 1 mM BCG achieved the most rapid increase in *A*628 when exposed to ammonia gas at each of the ammonia concentrations tested (11, 29, 100, and 239 ppm), and *A*628 reached a plateau within roughly 5 min on average (Figure 3a, see also below). Meanwhile, the sensors having BCG concentrations of 3, 5, and 10 mM required, respectively, roughly 20, 30, and 40 min on average to achieve plateaus of *A*628 upon exposure to gaseous ammonia at the tested ammonia concentrations (Figure 3b–d, see also below). These results showed that the time required to attain a saturation of *A*628 increased with increasing BCG concentration.

**Figure 4:**
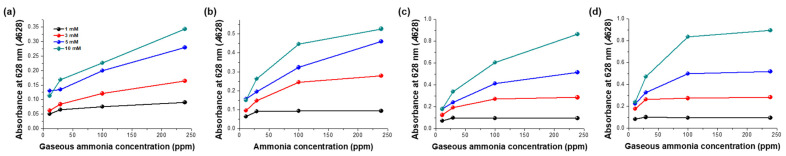
Levels of absorbance at 628 nm (*A*628) of colorimetric film sensors having different concentrations of bromocresol green (BCG) indicator and exposed to ammonia gas for (**a**) 1, (**b**) 3, (**c**) 5, and (**d**) 10 min.

For each colorimetric film sensor with a different concentration of the BCG indicator, we also plotted *A*628 versus gaseous ammonia concentration for each for various exposure times, namely 1, 3, 5, and 10 min (Figure 4). The *A*628 values of the sensors having BCG concentrations of 1 and 3 mM only marginally increased with increasing concentration of gaseous ammonia (black and red circles of Figure 4). In contrast, the sensors containing BCG concentrations of 5 and 10 mM each achieved significant increases in *A*628 with increasing concentration of the ammonia (blue and green circles of Figure 4). In particular, the *A*628 value of the sensor with 5 mM BCG and exposed to ammonia gas for 3 min increased sharply with increasing ammonia gas concentration, and did so linearly throughout the ammonia concentration range tested (blue circles of Figure 4b). Thus, an indicator concentration of 5 mM and an ammonia exposure duration of 3 min were chosen for achieving a sensitive detection of gaseous ammonia using the developed colorimetric film sensor, and were employed in the following experiments.

### 3.3. Validation of the Sensitivity and Selectivity of the Sensor Specific for Gaseous Ammonia

**Figure 5:**
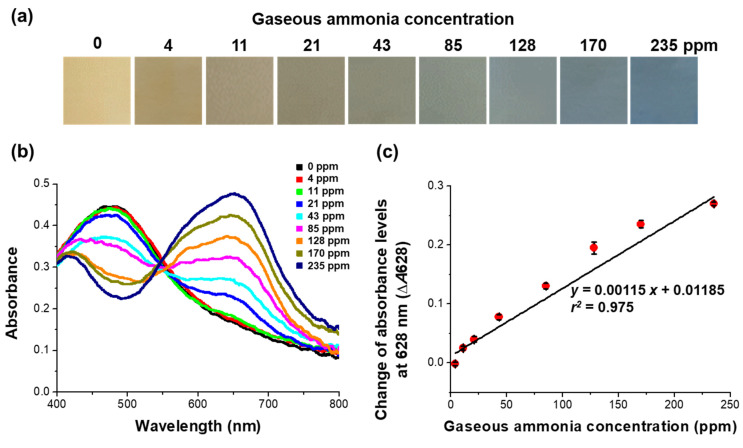
Sensitivity of the colorimetric film sensor for the detection of ammonia gas. (**a**) Optical images showing the colorimetric changes, (**b**) absorbance spectra, and (**c**) absorbance changes (Δ*A*628) of film sensors exposed to different concentrations of gaseous ammonia for 3 min.

The color of the colorimetric film sensor changed from yellow-orange to blue upon being exposed to gaseous ammonia for 3 min (Figure 5). The sensor exhibited an increasingly more distinct blue color with increasing concentration of ammonia gas, and color changes recognizable by the naked eye occurred as the ammonia gas concentration was increased throughout the 11–235 ppm range (Figure 5a). The changes in the visually observed color of the sensor corresponded to the changes in its absorbance spectrum (Figure 5b). The absorbance at 628 nm increased as the color of the sensor changed from yellow-orange to blue upon exposure to ammonia gas. The absorbance change (Δ*A*628) was found to be linearly proportional to the gaseous ammonia concentration, following the equation *y* = 0.00115 *x* + 0.01185 with *r*^2^ = 0.975 (Figure 5c). The sensitivity results demonstrated that our sensor was capable of achieving a simple and rapid detection of gaseous ammonia with a corresponding visual detection limit of 11 ppm. Note that the developed sensor, being colorimetric, was designed to be used for on-site workers requiring a visual confirmation of gaseous ammonia exposure. As the concentration of gaseous ammonia was increased to about 50 ppm, the blue color became stronger.

**Figure 6:**
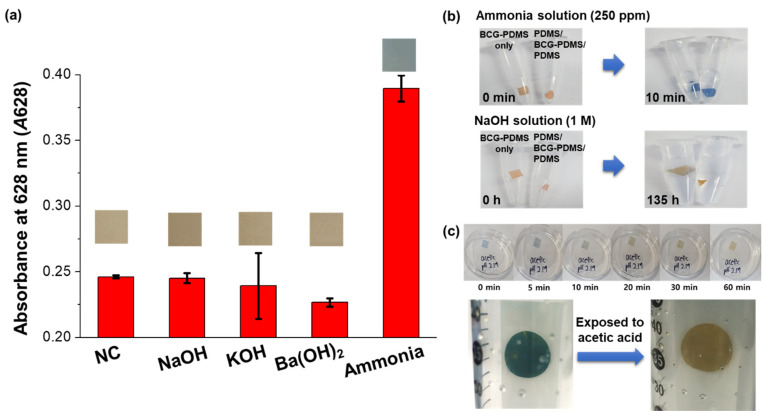
(**a**) Validation of the selectivity of the colorimetric sensor specific for gaseous ammonia. NC denotes negative control (no exposure to any chemicals). Insets show photographs of the sensors exposed to the indicated chemicals. (**b**) Optical images showing the changes in the colors of the BCG-PDMS film alone and the full PDMS/BCG-PDMS/PDMS sensor resulting from their immersions into ammonia and NaOH solutions. (**c**) Optical images showing the reversibility of the colorimetric sensor upon its exposure to the fume of acetic acid.

The colorimetric change of the film sensor was specific for gaseous ammonia (Figure 6a). The color of the sensor only marginally changed upon exposure to either NaOH, KOH, or Ba(OH)_2_, compared to the negative control (no exposure to any of the chemicals). The sensor became blue only when exposed to gaseous ammonia. As mentioned above, top and bottom protection films were employed to prevent a direct contact of the BCG indicator with aqueous base substances in the sample. Neither BCG-PDMS alone nor the full PDMS/BCG-PDMS/PDMS sensor showed any visually observable significant change in color upon being immersed for 9 h in a solution of 1 M NaOH. Upon 135 h of incubation, a slight color change was observed for the BCG-PDMS film only, i.e., with no protection layers (bottom photographs of Figure 6b), due to the direct contact of the aqueous hydroxide ions with BCG indicator in this case. On the other hand, both BCG-PDMS alone and the full PDMS/BCG-PDMS/PDMS sensor showed a rapid change in color to blue, specifically within 10 min, upon being exposed to a solution of 250 ppm of ammonia (top photographs of Figure 6b), because ammonia could effectively penetrate into both films. Note that insignificant changes in the color of samples of the full PDMS/BCG-PDMS/PDMS sensor occurred upon their being immersed in aqueous solutions of NaOH, KOH, and Ba(OH)_2_ (data not shown). In addition, the film sensor did not show any changes in color and absorbance spectra upon being exposed to the major atmospheric gases, specifically air, nitrogen, oxygen, hydrogen, or argon gases, indicating that the film sensor was not influenced by the ambient gases (Figure S2).

Our sensor was also found to be reproducible. It reverted from blue when exposed to just ammonia to its original color yellow-orange upon being subsequently exposed to the fume of acetic acid (Figure 6c) or to aeration for 48 h (date not shown). The reproduced sensor showed the reliable performance for the detection of gaseous ammonia as did the originally fabricated sensor, and the film sensor could be used at least five times (Figure S3). This reversibility of our sensor would contribute to it providing a low-cost and low-power alternative for simple and rapid recognition of gaseous ammonia, features particularly important in industrial settings. Moreover, any transformations and damages were not observed in the film sensor for an extended period of 2 years. The absorbance spectrum of the film sensor stored under ambient conditions for 2 years (green line of Figure S4) was similar to the spectrum of the original film sensor (black line of Figure S4), indicating the long-term stability of the film sensor (Figure S4).

Of the compounds tested, only gaseous ammonia was able to react with the BCG indicator in the middle layer of the sensor, by penetrating through the surrounding PDMS layers; in contrast, aqueous alkaline solutions were apparently unable to pass through the PDMS layers, due to the hydrophobic characteristics of the PDMS films [29,30]. This gas-diffusion process allowed selective detection of gaseous ammonia and excluded nonvolatile and particulate interferences. Colorimetric methods using various indicators have been reported for the detection of ammonia gas [33,34,35,36]. Cho et al. presented a paper sensor for the detection of dissolved ammonia [33]. The colorimetric response of the paper sensor was reported to be acquired by evaporating dissolved ammonia into the headspace of a closed sample vessel. This evaporation process provided selective detection of ammonia by avoiding interferences from nonvolatiles and particulates in the water samples, but it required a detection duration of 10 min. Advanced techniques have achieved superior sensing performances with low detection limits, rapid responses, and short recovery times [17,37]. A paper-substrate-based electrochemical sensor of ammonia gas was reported to exhibit a detection limit of 1 ppm and rapid response of 8 s [38]. Seekaew et al. reported a highly selective ammonia gas sensor based on tin−titanium dioxide/reduced graphene/carbon nanotube (Sn-TiO_2_@rGO/CNT) nanocomposites [39]. The gas sensor was reported to show a change in resistance upon being exposed to ammonia vapor in the concentration range of 25–250 ppm. Despite their excellent sensing performances, the use of advanced ammonia sensors typically requires expertise in performing the techniques and operating the instruments. In this regard, our colorimetric film sensor can be an alternative for affordable detection of ammonia gas with the advantage of simplicity.

### 3.4. Application of the Sensor for the Detection of Released Ammonia Gas

**Figure 7:**
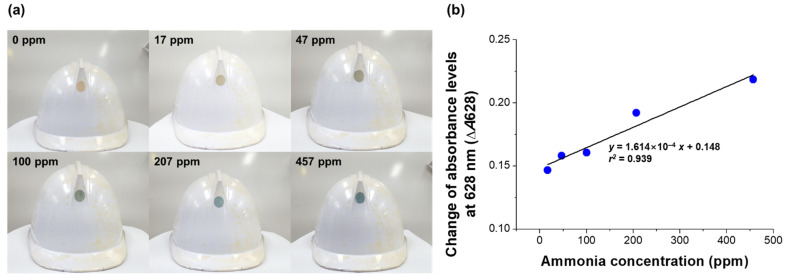
Application of the colorimetric film sensor onto a safety helmet for the detection of released ammonia gas. (**a**) Photographs and (**b**) absorbance changes (Δ*A*628) of attached film sensors exposed for 3 min to the various indicated concentrations of released ammonia gas.

As a simulation of ammonia gas leakage in an industrial setting, a colorimetric film sensor was employed on a safety helmet (Figure 7). The sensor was attached strongly onto the surface of the helmet and the original yellow-orange color of the sensor was clearly discerned by the naked eye in this setting (Figure 7a). The helmet with the attached sensor was placed in a chamber, and upon exposure of the helmet to ammonia gas released into the chamber, the attached sensor became blue within 3 min (Figure 7a). The sensor was found to be dark yellow-orange, and its edge began to become blue when it was exposed to low concentrations of ammonia gas, namely 17 and 47 ppm. The blue color of the film sensor became distinct upon exposure to higher concentrations of released ammonia gas, specifically in the range of 100–457 ppm.

The absorbance change (Δ*A*628) of the sensor attached to the helmet also increased upon its exposure to increasing concentrations of the released ammonia gas, and did so in a linear fashion following the equation *y* = 1.614 ×10^−4^ *x* + 0.148 and *r*^2^ = 0.939 (Figure 7b). The linear regression was different from that of the sensitivity validation (Figure 5c), with this difference attributed to the different operating conditions. The sensitivity test was operated in the batch system; thus, the film sensor was homogeneously exposed to the ammonia gas. Unlike the batch test used for the sensitivity validation, in the test with the sensor attached to the helmet, ammonia gas was introduced into the chamber by releasing it to simulate a leakage of gaseous ammonia in industries. The release of ammonia gas might lead to the heterogeneous distribution of ammonia gas in the chamber system. The different exposures probably resulted in the different linearities of absorbance change versus concentration of ammonia gas (Figure 5c and Figure 7b).

## 4. Conclusions

We demonstrated a successful development of a PDMS film-based colorimetric sensor suitable for detecting ammonia gas in air. Combining three layers into the film sensor was found to offer advantages of nonleachability, stretchability, reproducibility, and scalability. The developed sensor is fit for on-site workers in industries requiring facile detection of gaseous ammonia at concentration levels above those acceptable according to OSHA—as gaseous ammonia leakage was rapidly recognized by the naked eye without expertise in techniques and expensive instruments. Moreover, the simplicity and reproducibility of the sensor reduce fabrication and detection expenses, and, hence, provide an affordable option for small companies. We envision that the simple, rapid, cost-effective process we developed for fabricating the colorimetric sensor of ammonia can be further extended to the food packaging industry, specifically for meat and fish, as significant levels of total volatile nitrogen compounds can be produced during the processing, storage, and transport of such food.

## Supplementary Materials

The following are available online at https://www.mdpi.com/article/10.3390/bios12080664/s1, Figure S1: Scanning electron microscope (SEM) images of the colorimetric film sensor. Figure S2: (**a**) Photographs and (**b**) absorbance spectra of film sensors upon exposure to gases, specifically air, argon (Ar), oxygen (O2), nitrogen (N2), hydrogen (H2), and ammonia (NH3). Figure S3: Reusability of the colorimetric film sensor. Changes of absorbance at 628 nm (*A*628) of the sensor reused five times are presented. Figure S4: Stability of a colorimetric film sensor. Absorbance spectra of the film sensor before (i.e., original film sensor, black line) and after stored at ambient condition for 1 (red line) and 2 years (green line), respectively.
