# Supplementary material for: A Flexible and Attachable Colorimetric Film Sensor for the Detection of Gaseous Ammonia
Source: Biosensors (Basel). 2022 Aug 21;12(8):664. doi: 10.3390/bios12080664 (PMC9405545; doi:10.3390/bios12080664)
Supplement: Supplementary file 1 [file biosensors-12-00664-s001.zip › biosensors-1849968-supplementary.pdf]

Supplementary Information

A Flexible and Attachable Colorimetric Film Sensor for the Detection of Gaseous Ammonia

Sangwon Lee [1], Eun-Hee Lee [2,*] and Seung-Woo Lee [1,3,4,*]

[1] Department of Fine Chemistry, Seoul National University of Science and Technology, 232 Gongneung-ro, Nowon-gu, Seoul, 01811, Korea
[2] Department of Microbiology, Pusan National University, 2 Busandaehak-ro 63 Beon-gil, Geumjeong-gu, Busan, 46241, Korea
[3] Department of Nano Bio Engineering, Seoul National University of Science and Technology, 232 Gongneung-ro, Nowon-gu, Seoul, 01811, Korea
[4] Center for Functional Biomaterials, Seoul National University of Science and Technology, 232
Gongneung-ro, Nowon-gu, Seoul, 01811, Korea
* Correspondence: leeeh@pusan.ac.kr (E.-H.L.); swlee@seoultech.ac.kr (S.-W.L.)

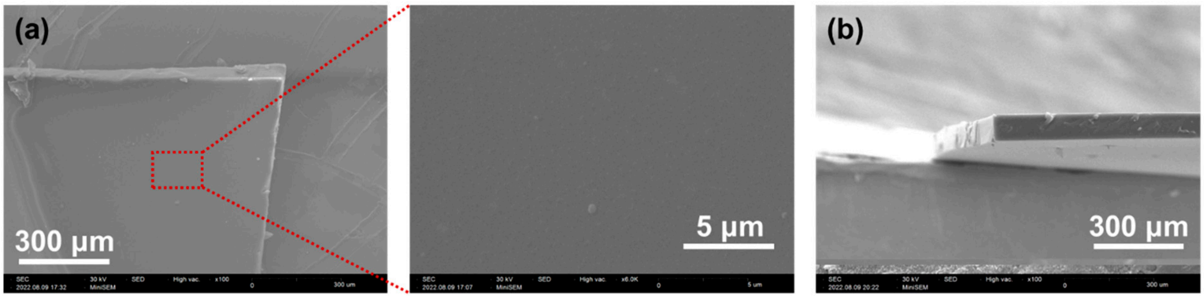

Figure S1. Scanning electron microscope (SEM) images of the colorimetric film sensor. (a) Surface and (b) cross-sectional images of the sensor.

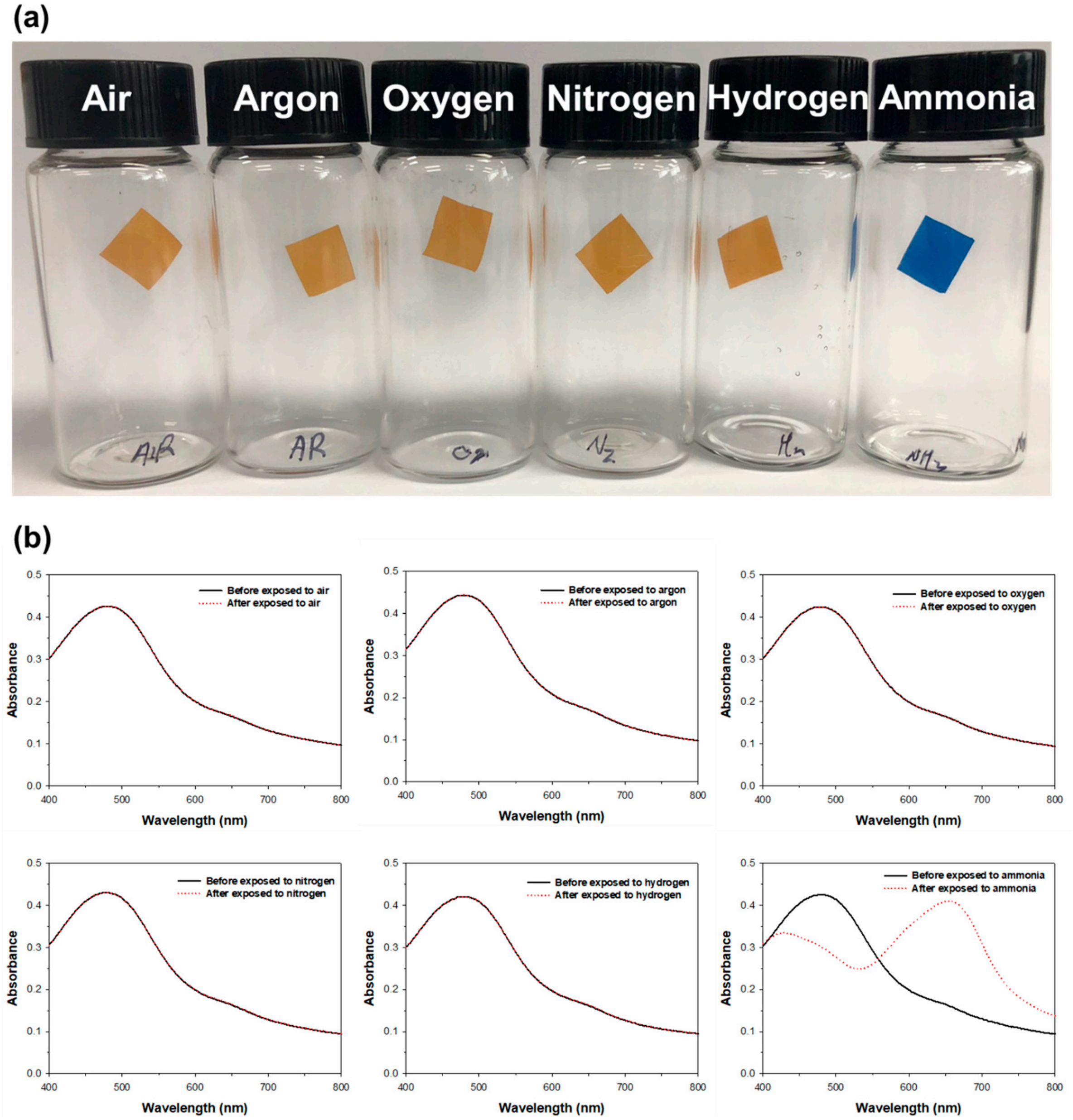

Figure S2. (a) Photographs and (b) absorbance spectra of film sensors upon exposure to gases, specifically air, argon (Ar), oxygen (O_2), nitrogen (N_2), hydrogen (H_2), and ammonia (NH_3).

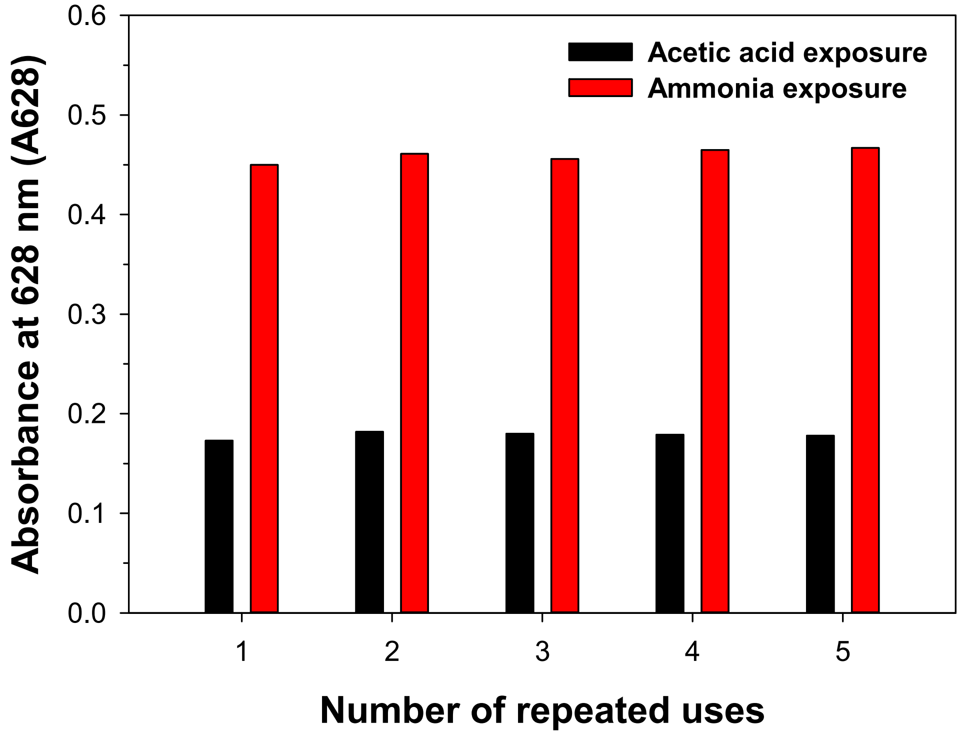

Figure S3. Reusability of the colorimetric film sensor. Changes of absorbance at 628 nm (A628) of the sensor reused five times are presented.

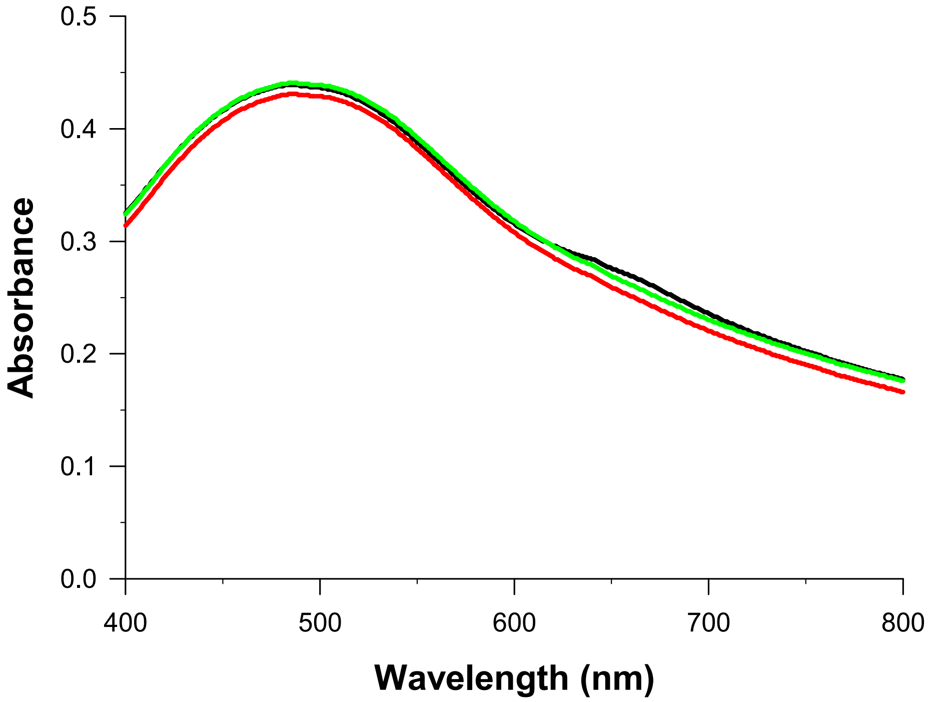

Figure S4. Stability of a colorimetric film sensor. Absorbance spectra of the film sensor before (i.e., original film sensor, black line) and after stored at ambient condition for 1 (red line) and 2 years (green line), respectively.
